# Atoh8, a bHLH Transcription Factor, Is Required for the Development of Retina and Skeletal Muscle in Zebrafish

**DOI:** 10.1371/journal.pone.0010945

**Published:** 2010-06-03

**Authors:** Jihua Yao, Jingyao Zhou, Qiaoling Liu, Daru Lu, Lu Wang, Xiaojing Qiao, William Jia

**Affiliations:** 1 State Key Laboratory of Genetic Engineering, Institute of Genetics, School of Life Sciences, Fudan University, Shanghai, China; 2 Pathology Laboratory, Shanghai Research Center for Biomodel Organisms, Shanghai, China; 3 Department of Surgery, University of British Columbia, Vancouver, Canada; Texas A&M University, United States of America

## Abstract

**Background:**

*Math6/atoh8*, a bHLH transcription factor, is thought to be indispensable for early embryonic development and likely has important roles in vertebrate tissue-specific differentiation. However, the function of Atoh8 during early development is not clear because homozygous knockout causes embryonic lethality in mice. We have examined the effects of the *atoh8* gene on the differentiation of retina and skeletal muscle during early development in zebrafish.

**Results:**

We isolated a *Math6* homologue in zebrafish, designated as zebrafish *atoh8*. Whole -mount *in situ* hybridization analysis showed that zebrafish *atoh8* is dynamically expressed mainly in developing retina and skeletal muscle. Atoh8-MO knock-down resulted in reduced eye size with disorganization of retinal lamination. The reduction of *atoh8* function also affected the arrangement of paraxial cells and differentiated muscle fibers during somite morphogenesis.

**Conclusion:**

Our results show that Atoh8 is an important regulator for the development of both the retina and skeletal muscles necessary for neural retinal cell and myogenic differentiation during zebrafish embryogenesis.

## Introduction

Basic helix-loop-helix (bHLH) genes control both the initial neuronal fate determination and subsequent neuronal differentiation/maturation during neurogenesis [1]. Previous studies have suggested that several neurogenic bHLH genes such as *Mash1, Math3* and *Neurogenin* facilitate neuronal fate determination, rather than the glial cell fate [2], [3]. In contrast, bHLH genes *Hes1, Hes5* and *Hesr2* functionally antagonize neurogenic bHLH genes and promote gliogenesis[4]. Misexpression of bHLH genes in murine cerebral cortex was shown to affect cell fate choices and neuronal survival [5]. In mouse retina, glial cell fate specification is modulated positively by the bHLH gene *Hes5* [4], [6]. bHLH genes such as *NeuroD* and *Math2 (Nex1)* are expressed in differentiating neurons after neuronal fate determination. Mutations of these genes in neurons cause defects in terminal differentiation/maturation [3].

*Atoh8*, a novel member of the bHLH gene family, has an obvious structural similarity to the *Drosophila* proneural gene *atonal* [7]. The human Atoh8 contains 321 amino acids with one bHLH domain. Atoh8 might be a transcriptional activator and a permissive factor for terminal podocyte differentiation [8]. It was implicated in differentiation of neuronal cell lineages in the brain by the observation that overexpression of *atoh8* in the developing retina induced neurogenesis while inhibiting gliogenesis [9]. Recently, it has been found that Atoh8 may also be involved in the development of kidney [8], liver [10] and pancreas [11].

In the present study, we cloned and characterized the zebrafish *atoh8* gene. In addition, we investigated the distribution and time course of the expression of zebrafish *atoh8* with special attention paid to the role of this gene in the development of the retina and somites of embryonic zebrafish.

## Results

### Structure of the zebrafish *atoh8* gene

**Figure 1:**
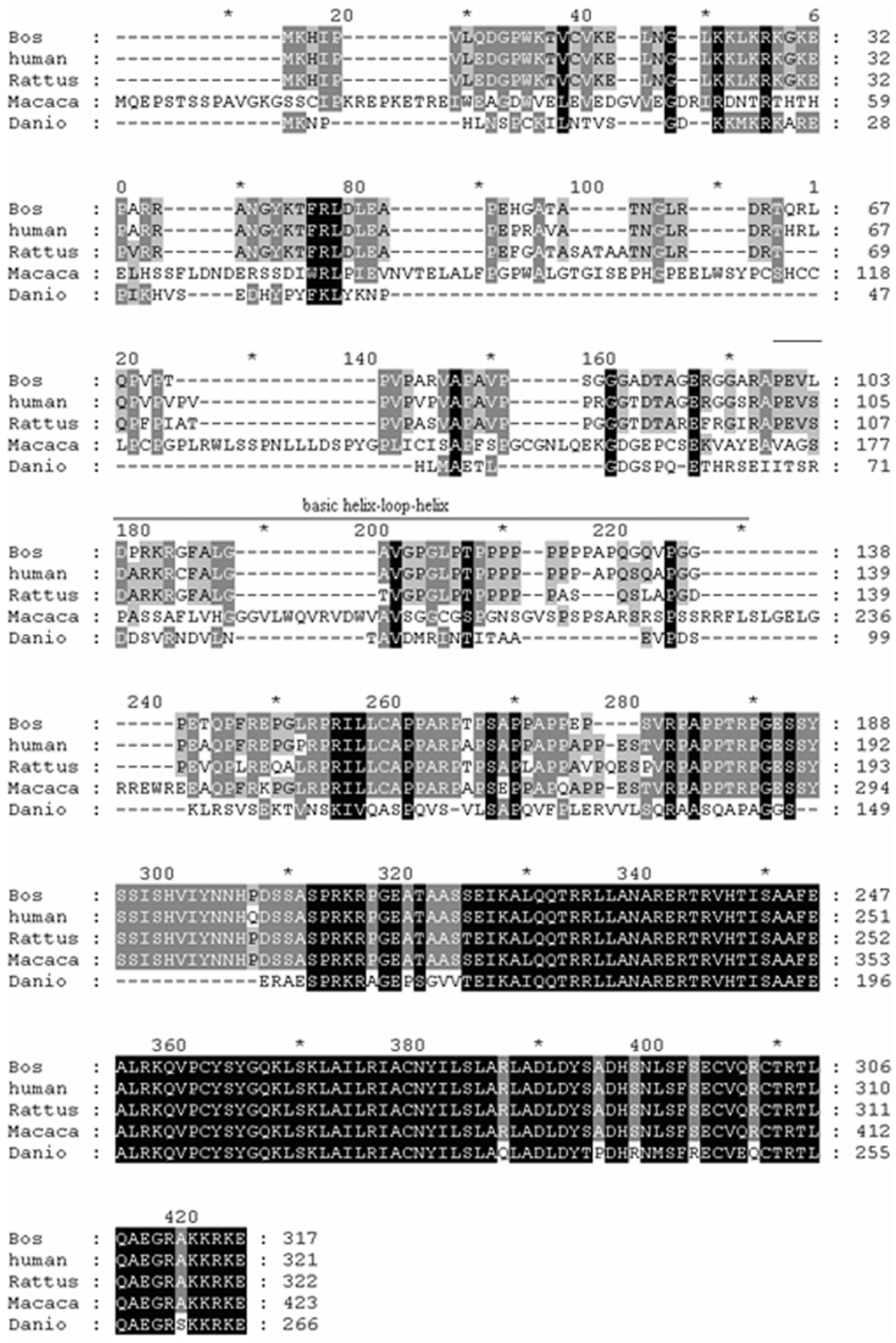
Alignments of bovine, human, rat, monkey and zebrafish Atoh8. Amino acids are shaded according to the degree of conservation: black (100% similarity), gray (80% similarity) and light gray (60% similarity). The amino acid sequences of *Bos taurus* (Bovin), *Homo sapiens* (Human), *Rattus norvegicus* (Rat), *Macaca mulatta* (Monkey) and *Danio rerio *(Zebrafish) were obtained from the GenBank database with Accession Nos. XP_873292, NP_116216, NP001102711, XP_001084825 and EU272033, respectively.

The zebrafish *atoh8* genomic sequence spans 13872bp containing 3 exons and 2 introns. The ORF of *atoh8* cDNA is 873 bp (Accession No. EU272033) and encodes a putative protein of 266 amino acids which shares 52%, 51%, 50% and 46% amino acids sequence identity with the orthologues from *Bos taurus* (XP_873292), *Homo sapiens* (NP_116216), *Rattus norvegicus* (NP001102711), and *Macaca mulatta *(XP_001084825), respectively. The alignment of the bovine, human, rat, monkey and zebrafish Math6/Atoh8 factors is shown in Figure 1.

### Zebrafish *atoh8* is highly expressed in retina and skeletal muscle

**Figure 2:**
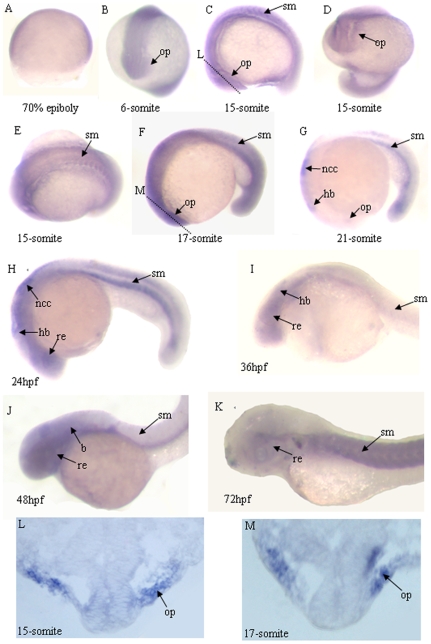
Expression patterns of the zebrafish *atoh8* gene during morphogenesis. Lateral view, anterior to the left (A,C,F–K). Top view (B,D). Dorsal view, anterior to the left (E). (A) 70% epiboly. (B) 6-somite. (C–E) 15-somite. (F) 17-somite. (G) 21-somite.(H,M,N) 24 hpf. (I) 36 hpf. (J) 48 hpf. (K) 72 hpf. (L–M)horizontal section of retina from 15-somite to 17-Somite stage embryos. *Abbreviations*: *b, brain*; hb, hind brain; *lp, lens primordium;ncc,neuron crest cell; op, optic vesicle*; *re, retina; sm, somite*.

To gain insights into the role of the zebrafish *atoh8* gene, we analyzed its spatiotemporal expression pattern during embryo development using whole-mount *in situ* hybridization (Figure 2). The results showed that zygotic *atoh8* mRNA is expressed at as early as epiboly stage, and is broadly distributed till early segmentation stage (Figure 2A–B). Later, the signals are expressed dynamically and mainly in developing retina and skeletal muscle (Figure 2A–M).

In the eye, *atoh8* expression was detected in the optic primordium at the 6-somite stage when the optic vesicle is first distinguished (Figure 2B). *Atoh8* was steadily expressed during the invagination of the optic vesicle from its initiation to its expansion to the lens rudiment (Figure 2C–F, 2L–M).

In skeletal muscle, a faint *atoh8* signal could be detected in muscle precursor cells at the early segmentation period (Figure 2B–F), and staining of ventral somites was visible from the 21-somite to 24 hpf (hour post fertilization) stage (Figure 2G–H). *Atoh8* expression had decreased sharply during 36 to 48 hpf (Figure 2I–J) while it reached its highest level at about 72 hpf (Figure 2J–K). During this period, the distribution of *atoh8* extended to the dorsal somites (Figure 2K). In addition, the *atoh8* signal also appeared in the brain and neural crest cell (Figure 2G–J).

### Knock-down of Atoh8 influenced embryonic development

**Figure 3:**
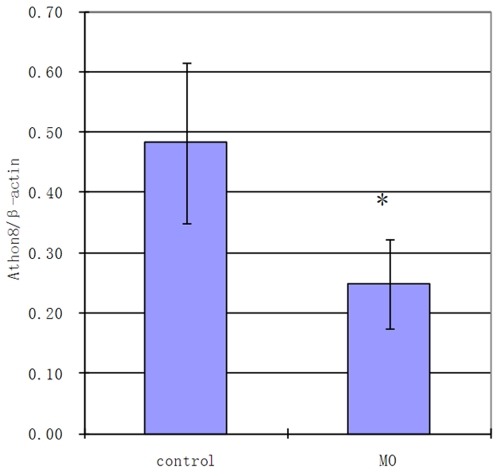
Amount of mature transcript of *atoh8* in splicing blocking morpholino (MO2) treated zebrafish embryos by qRT-PCR. The MO was targeting the splicing region between intron 1 and exon 2 and significantly reduced levels of *atoh8* mRNA. Two-tail student t test, * p<0.05.

**Figure 4:**
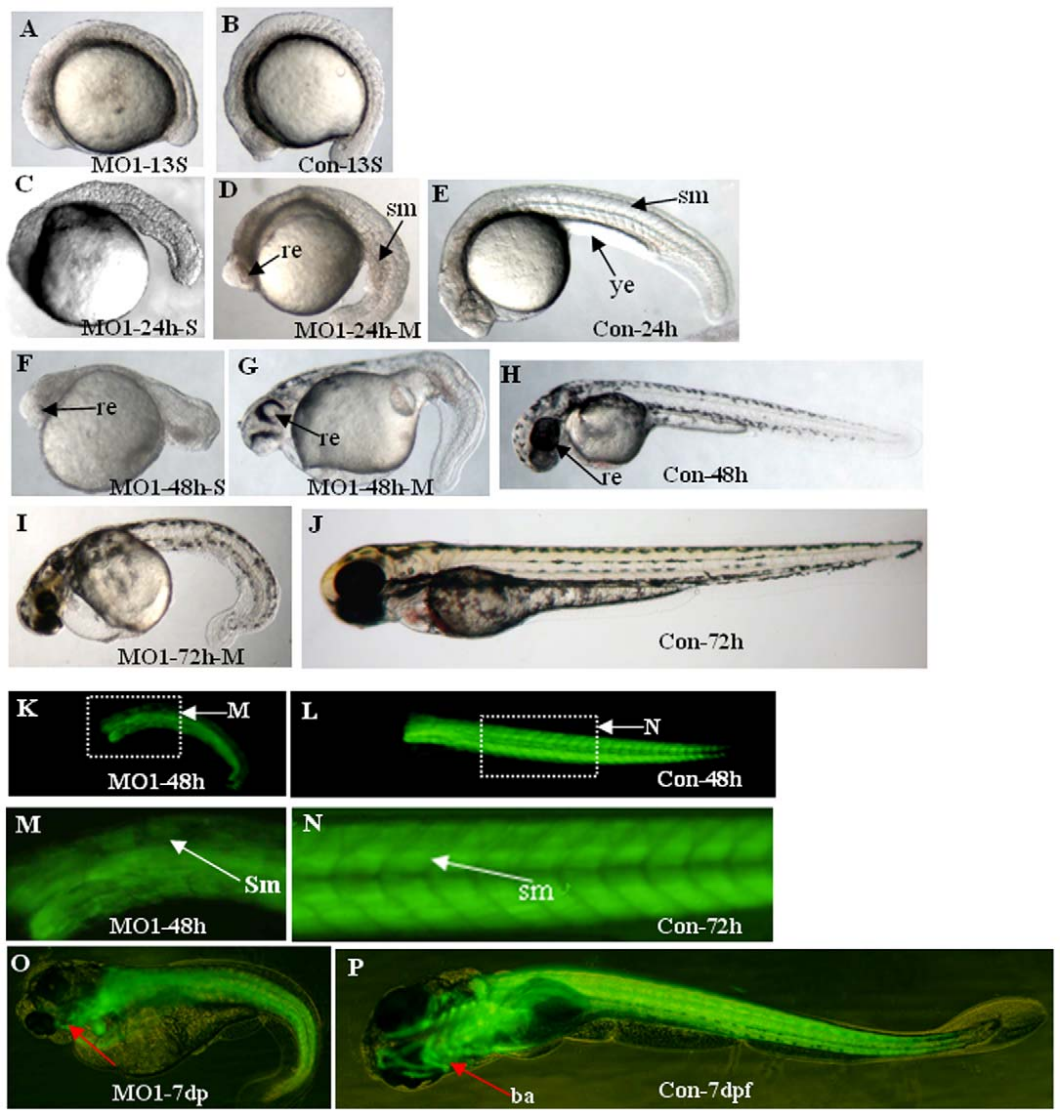
Phenotypes of translation blocking morpholino (MO1) knockdown embryos. (A–B)13-somite stage. (C–E) 24 hpf. (F–H) 48 hpf. (I–J) 72 hpf. (K–N) somite of mylz2-GFP transgenic zebrafish embryos at 48 hpf. (O–P) mylz2-GFP transgenic zebrafish embryos at 7dpf. A, C–D, F–G, I, K, M and O are Atoh8-MO treated embryos: A, C and F show severe abnormality, D, G, I, K, M and O show mild abnormality, and B, E, H, J, L, N and P are control-MO treated embryos. Atoh8-MO-treated embryos show shrunken eyes and head, a curved body axis with indistinct somite boundaries and yolk extension, accompanied by a U-shaped somite formation. In addition, the branchial arches are greatly reduced(O–P). In all panels but P, the dorsal view is up and the anterior is to the left. P shows the ventral view and the anterior to the left. *Abbreviations*: *re, retina; sm, somite; ba*, *branchial arches*.

**Figure 5:**
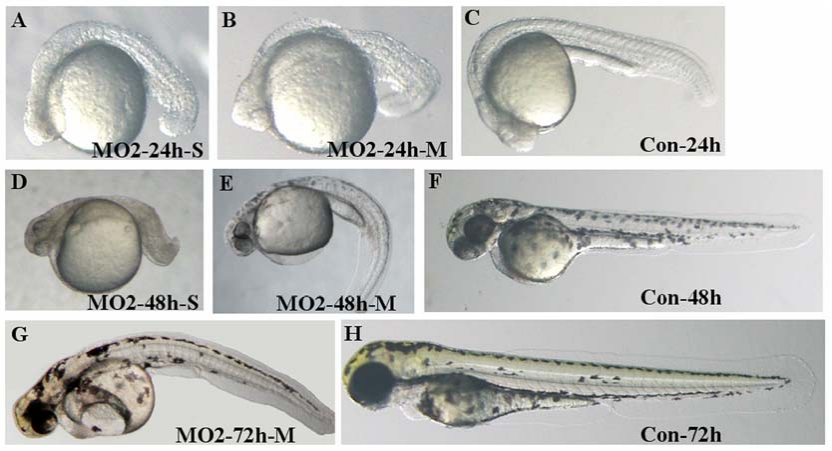
Phenotypes of splicing blocking morpholino (MO2) knockdown embryos. (A–C) 24 hpf. (D–F) 48 hpf. (G–H) 72 hpf. A–B, D–E and G are MO2 treated embryos: A and D, severe abnormality, B, E and G, mild abnormality and C, F and H, control-MO treated embryos. The figure shows that splicing blocking morpholino treated embryos show similar phenotypes as that of translation blocking MO1-treated ones.

**Figure 6:**
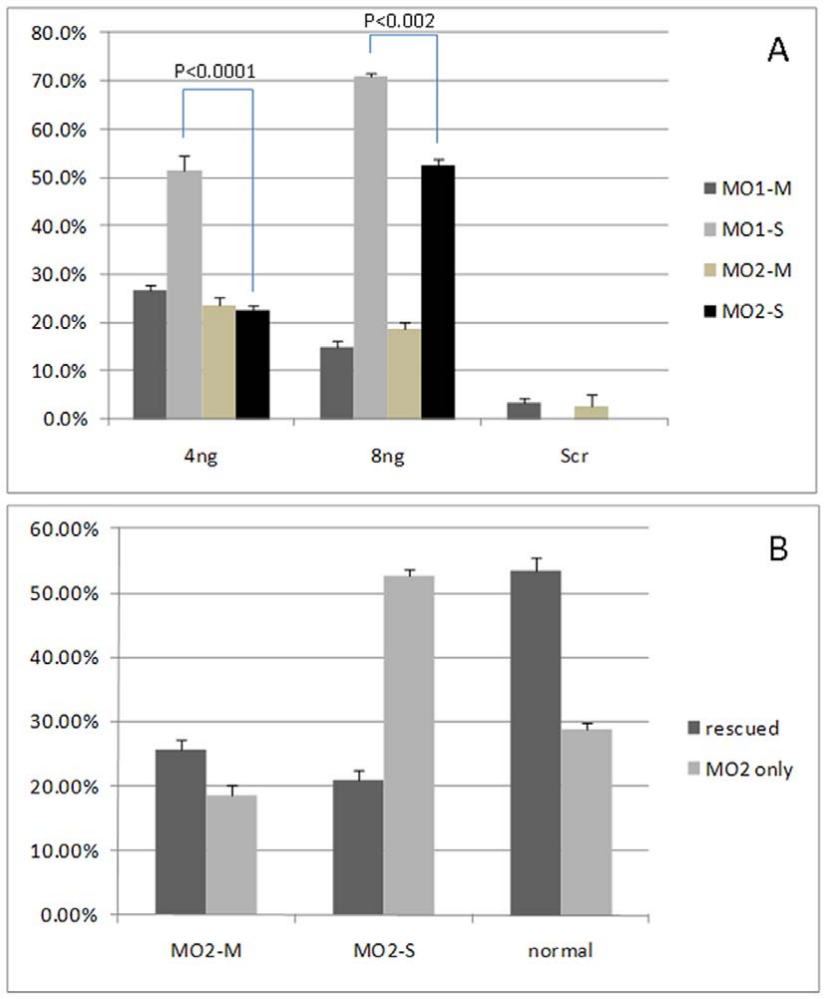
Percentages of abnormal embryos in morpholino treated with or without co-expression of exogenous Atoh8 mRNA. A: Percentages of abnormal animals with mild or severe phenotypes following the injection of 4 ng or 8 ng morpholinos. MO1-M and MO1-S, percentages of animals with mild and severe phenotypes followed injection with translation blocking morpholino MO1 (total injected n = 169 for 4 ng MO1 and n = 182 for 8 ng MO1), respectively. MO2-M and MO2-S, percentages of animals with mild and severe phenotypes followed injection with splicing blocking morpholino MO2 (total injected n = 173 for 4 ng MO2 and n = 192 for 8 ng MO2), respectively. MIS-MO, morpholino with mismatched sequences (n = 154 for MIS-MO1, and n = 114 for MIS-MO2, 8 ng each). The data were shown by the averages of three independent experiments for each group. B. Overexpression of *atoh8* mRNA prevents the severe form of developmental defect in splicing blocking morpholino (MO2) injected embryos (n = 140 for MO2 rescue and n = 192 for MO2 alone, respectively). Two-tail Student t test, * p<0.05, ** p<0.0001.

**Figure 7:**
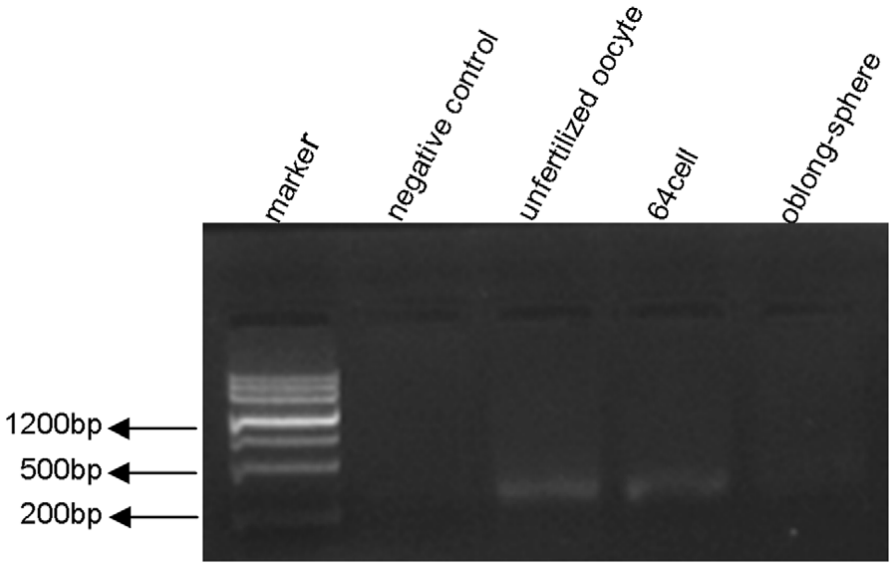
RT- PCR detection of maternally supplied *atoh8* mRNA in zebrafish. Maternally supplied *athoh8* mRNA was detected by RT-PCR in both unfertilized oocytes and 64-cell stage but significantly reduced in oblong-spheres. The negative control contains no sample template.

To further investigate *atoh8* function in the development of the retina of zebrafish, two morpholinos (MOs) were designed., Atoh8*-*MO1 (MO1) and Atoh8splI1E2 (MO2), targeting the translation starting point and the splicing between intron 1 and exon 2 region of *atoh8*, respectively. Quantitative RT-PCR showed that MO2 significantly reduced (p<0.05) levels of matured *atoh8* mRNA by approximately 50% (Figure 3). Both MOs dose dependently caused developmental deficiency in zebrasifh embryos (Figure 4, 5 and 6A) shown by two phenotypes, mild type and severe type. The severe phenotype displayed a shrunken head, reduced eye size and fused somites (Figure 4A, C, F and Figure 5A, D). Most embryos with this phenotype died within 3 days. On the other hand, embryos with the mild phenotype displayed an abnormal brain shape with smaller eyes, a curved body axis with indistinct somite boundaries and frequently reduced or no circulation (Figure 4D,G,I,K,M,O and Figure 5B, E, G). In addition, the yolk extension was hardly visible starting at the beginning of tail straightening from the 17-somite stage (Figure 4C, D, F, G, I and Figure 5A, B, D, E, G). The tail also appeared to be less flexible than that of the controls. In addition, the branchial arches were greatly reduced (Figure 4O). Knock-down of Atoh8 in mylz2-GFP transgenic zebrafish expressing skeletal-muscle-specific GFP provide more evidence for the effect (Figure 4K–P). The translational blocking morpholino (MO1) caused 51.5±2.9% and 70.9±0.75% severe as well as 26.6±1.2% and 14.8±1.3% mild deficient phenotypes at the concentrations of 4 ng/embryo and 8 ng/embryo, respectively. The splicing blocking morpholino (MO2) caused 22.5±1.1% and 52.6±1.2% severe as well as 23.7±1.5% and 18.7±1.4% mild deficient phenotypes at the concentration of 4 ng/embryo and 8 ng/embryo, respectively. It is interesting to note that treatment with the two MOs caused similar percentages of mild deficiency phenotype but the translation blocking morpholino (MO1) resulted in much higher rate of severe type than the splicing blocker (MO2) (p<0.0001 for 4 ng groups and p<0.002 for 8 ng groups, Figure 6A). While it is possible that MO1 may have higher non-specific toxicity than MO2, another explanation is that some *atoh8* transcripts are maternally supplied. To investigate this possibility, RT-PCR was conducted on total RNAs collected from unfertilized eggs and embryos of 64-cell stage as well as oblong-sphere stage. As shown in Figure 7, maternally supplied *atoh8* mRNA was evidently present in both unfertilized oocytes and 64-cell stage but not the oblong-spheres, indicating preexisting of *atoh8* mRNA that was not affected by the spicing blocker MO2 but was prevented from protein synthesis by the translation blocker MO1.

To further verify the specificity of above Atoh8-MOs caused abnormality, 200 pg mRNA coding *atoh8* cDNA was co-injected with 8 ng splicing blocker morpholino MO2 into the embryos (n = 140). Compared to animals injected with 8 ng MO2 alone (n = 192), the percentage of normal embryos was substantially increased in mRNA injected group (53.5±2.1% vs 28.7±1.2%, p<0.0001) (Figure 6B). Interestingly, while there was a slight increase of mild deformed embryos in mRNA co-injected embryos versus *atoh8* knockdown ones (25.6±1.5% vs. 18.7±1.6%, p<0.05), injection with the mRNA dramatically rescued embryos from severe deficiency (20.8±1.8% vs 52.6±1.2%, p<0.0001).

To rule out the possibility that the effect of Atoh8-MO was mediated through a general p53-induced apoptosis [12]–[13], all Atoh8-MOs were co-injected with a p53-MO (at a concentration ratio of 1∶1.5). Furthermore, there were no differences *in atoh8* morphants with or without p53-MO demonstrated by a separate experiment (data not shown). Thus, the abnormality in zebrafish development caused by Atoh8 knock-down is probably independent of p53 activity.

### Atoh8 is required for retinal lamination

**Figure 8:**
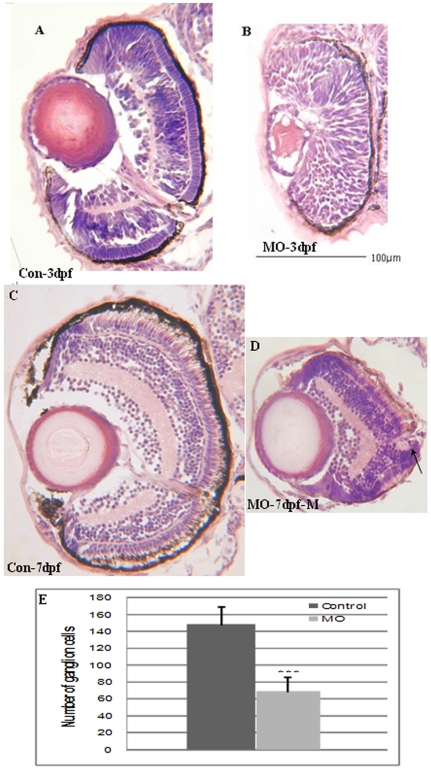
Transverse sections of Atoh8-MO treated embryo retina. (A,C) Control eyes at 3dpf and 7dpf, respectively. (B,D) Eyes of MO-treated fish at 3dpf and 7dpf, respectively. The section shows that the retina did not develop normal neuronal stratification compared with the control at 3dpf. By 7pdf, lamination had formed in the treated embryos, however, the retinal size was remarkably reduced (D), and the number of retinal ganglion neurons was significantly reduced in MO fish (E). (p<0.001). Besides, the photoreceptor layer of the morphant was less elongated. In all panels, the ventral is down.

To clarify the role of Atoh8 during retinal development, we examined a series of tissue cross-sections of the retinas of Atoh8 morphants that were treated with MO1 and survived for 3dpf and 7dpf. As shown in Figure 8, neuronal stratification had not appeared in the Atoh8 knock-down retina by 3dpf when retinal lamination in the control animals was already well developed (Figure 8A–B). By 7 dpf (Figure 8C–D), although lamination had formed in retinas of the treated embryos, the neural retina was significantly underdeveloped. The number of retinal ganglion cells was reduced by approximately 50% in *atoh8* knock-down animals (Figure 8E, 69.8±17.0 vs.149±21.1, n = 8, p<0.001). Moreover, the photoreceptor cells of the morphants had a less elongated appearance compared with the control. In addition, the portion of the photoreceptor layer ventral to the optic nerve seemed immature with a group of cells appearing from the inner nuclear layer (Figure 8D, arrow).

**Figure 9:**
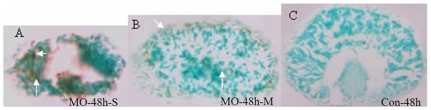
TUNEL assay of retina of Atoh8 knockdown embryos at 48hpf. (A) severely defective embryo retina shows serious cell apoptosis and (B) a mildly malformed embryo retina appears to contain only a minor population of apoptotic cells compared to the control (C). The brown cells were TUNEL-positive (arrow).

To determine whether apoptosis played a role in the malformation of the eye caused by Atoh8 knock-down, TUNEL staining was performed in 48 hpf embryos with or without MO treatment. There was a substantial number of apoptotic cells in the retina of morphants (Figure 9). More apoptotic cells were seen in severely mal-developed eyes than in those less affected by the Atoh8 knock-down.

**Figure 10:**
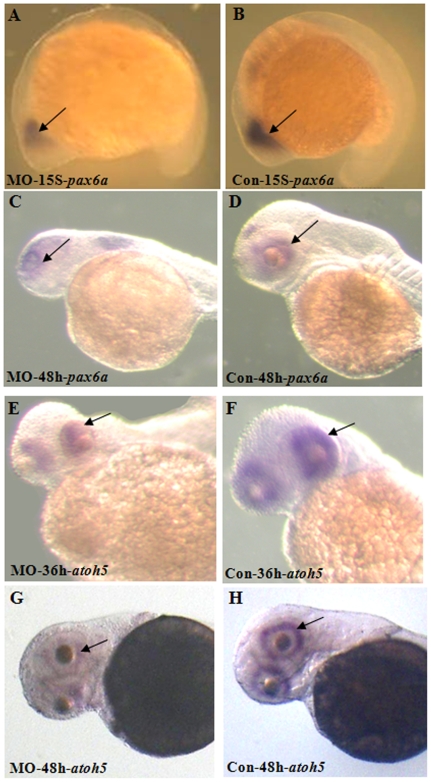
Retinal development is disrupted in Atoh8 knockdown embryos. (A–B) 15-Somite stage; (C–D,G–H) 48 hpf; (E–F) 36 hpf, (A,C,E,G) morphant and (B,D,F,H) control. Staining of *pax6a* showed that retinal region was remarkably reduced (arrow) in this MO-treated embryo. A*toh5* expression was present throughout most of the neural retinal at 36 hpf in the control embryo(E) while present only in dorsal and temporal retina in the MO-treated embryo(F). By 48 hpf, atoh5 localized throughout retina ganglion cells in the control(G) but present only in ventral retina in the MO-treated embryo(H). In addition, the signals of these two genes were much weaker than those of the control, indicating that knock-down of Atoh8 may have influenced retinal organization. In all panels, the dorsal side is up and the anterior is to the left.

Delayed retinal lamination in Atoh8 knock-down fish suggests the involvement of the gene in the fundamental mechanism of retinal development. Thus, we speculated that the levels of *atoh8* may regulate the expression of major eye development markers such as *Pax6* and *atoh5*. The *Pax6* gene is expressed throughout the development of vertebrate eyes and controls many aspects of ocular formation [14]–[15] while the *atoh5* gene is an earliest marker of neuronal production [16]. Transcriptional levels of both genes were measured in Atoh8 morphants. Both *Pax6* and *atoh5* were distributed in much smaller areas with a lower intensity of their signals in the Atoh8 knock-down embryos (Figure 10). Expression of *pax6a* in the retinal was remarkably reduced (arrow) in this MO-treated embryo (Figure 10A–D). *Atoh5* expression was present throughout most of the neural retinal at 36 hpf in the control embryo (Figure 10E) while present only in dorsal and temporal retina in the MO-treated embryos (Figure 10F). By 48 hpf, expression of *atoh*5 was localized in all retina ganglion cells in the control (Figure 10G) but present only in ventral retina in the MO-treated embryo (Figure 10H). In addition, the signal of the two genes were much weaker than those of the control, indicating that normal level of *atoh8* expression is required for adequate levels of both *Pax6* and *atoh5* in the developing retina.

### Atoh8 is important for myogenic differentiation of skeletal muscle

**Figure 11:**
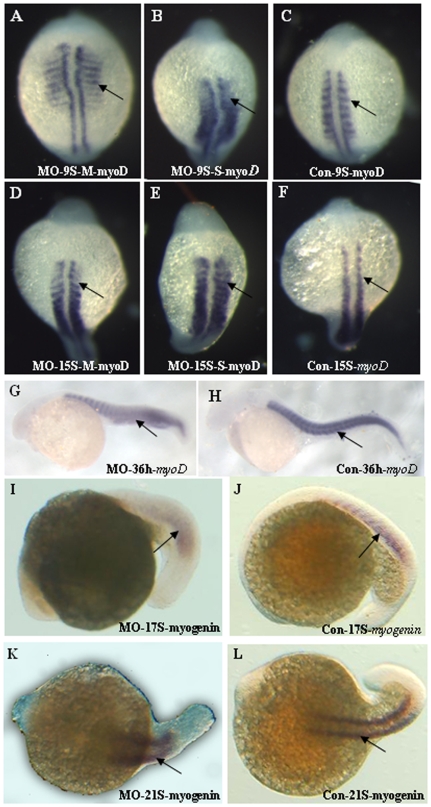
Skeletal muscle development is altered in Atoh8 knock-down embryos. (A–H) *myoD* in situ hybridization; (I–L) *myogenin* in situ hybridization. (A–C) 9-Somite stage; (D–E) 15-Somite stage; (G–H) 36 hpf; (C,F,H,J and L) are controls and the remainder are morphants with (B) and (E) the severely affected embryos. Expression of the skeletal marker myoD in paraxial mesoderm showed to tendency towards a ventral localization (A,D) or the boundaries of the posterior somites were indistinct(B,C) and a U-shaped somite formation appeared in the morphant (G). Expression of the skeletal marker myogenin provides further evidence of abnormality (I–L).

**Figure 12:**
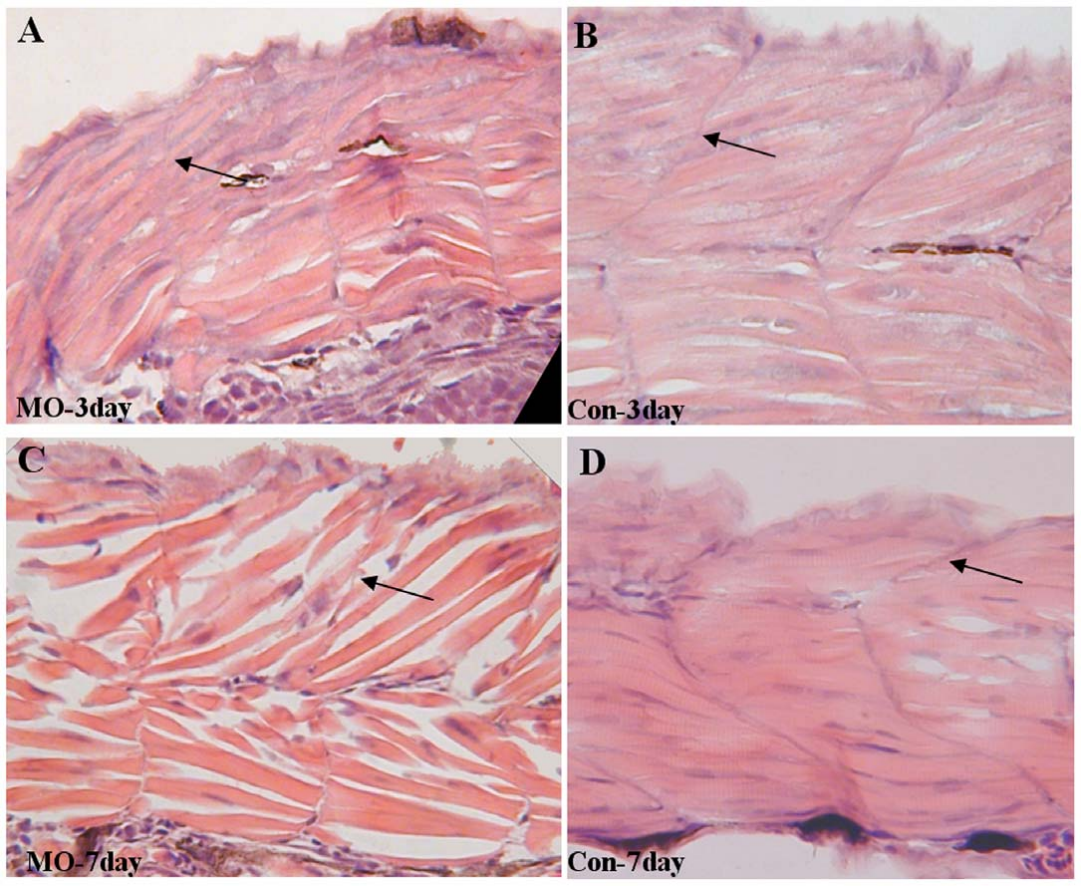
Absence of Atoh8 affects the myogenic differentiation of skeletal muscle. (A–B) transverse section of somites at 3dpf and (C–D) transferse section of somites at 7dpf. (A,C) somites of Atoh8-treated embryos; (B,D) the related controls. Note that the transverse myoseptum of MO-treated embryos is ill-defined and incomplete and that the somites form a U-shape or even appear cuboidal. Somites in controls, on the other hand, are chevron shaped with a distinct somite boundary. In addition, differentiated muscle fibers in myotomes show a less compact and orderly looking arrangement in the morphant.

To explore the function of *atoh8* in the development of muscle, we examined the expression of several skeletal muscle markers in Atoh8 knock-down embryos using whole-mount *in situ* hybridization. We found that the expression of myogenic myoD in paraxial mesoderm had a tendency toward a ventral localization (Figure 11A, G). By 36 hpf, the boundaries of the posterior somites were indistinct, even in the mildly abnormal embryos (Figure 11). Embryos with a severe deficiency showed fewer posterior somites in myogenin at the 17–21 somite stage than did the controls (Figure 11 I,K). The transverse myoseptum of Atoh8 MO-treated embryos was ill-defined and incomplete. Thus, the somites formed a U or even a cuboidal shape instead of the chevron shape of the control (Figure 12A–D). The malformation of somites in Atoh8 knock-down mylz2-GFP embryos was even more evident (Figure 4K–N). In addition, an examination of cross-sections of the embryos demonstrated that differentiated muscle fibers in myotomes were less compact and had a disordered arrangement (Figure 12A–D). Furthermore, the markedly reduced GFP signal in the knock-down embryos may also reflect a deficiency in the related structures including the skeletal muscle of somites and branchial arches (Figure 4K–P). Interestingly, unlike the retina, there was no detectable apoptosis in the muscle tissue of *atoh8* knock-down fish (data not shown). Taken together, these data support the idea that *atoh8* plays an important role in the organization of skeletal muscle during zebrafish development.

## Discussion

In this study, we isolated a zebrafish bHLH gene designated as *atoh8*, that is homologous to the mammalian *Math6* gene and the *Drosophila* proneural gene *atonal* [7]. We have shown that *atoh8* is expressed in retinas and somites with a pattern closely associated with neuronal differentiation and myoseptum formation. Severe malformation of the somites and eyes were seen in Atoh8 knockdown embryos, indicated by both abnormal morphology and reduced marker gene expression. In addition, reduced Atoh8 function resulted in an alteration of retinal structures with a significantly reduced number of ganglion neurons.

In the present study, MO knockdown of *atoh8* expression resulted in developmental deficiency. Both translation and splice blocking morpholinos gave rise to the same two phenotypes i.e. mild and severe abnormality. Since severe phenotype can be largely eliminated by overexpressing its mRNA, one cannot rule out that the mild phenotype may be caused by non-specific effect of the morpholinos. However, it is equally possible that the smaller difference in rates of mild abnormality between mRNA rescued and control groups was caused by part of severe phenotype becoming mild ones and the similar portion of mild type becoming normal type. More interestingly, there was a higher rate of severe phenotype by translation blocking morpholino than the splicing blocker. indicating the importance of maternally supplied *atoh8* transcript and fundamental functions of this gene in early embryonic development.

*Atoh8* is a member of the bHLH family that has been isolated from Drosophila, mosquitos, mice, rats and humans. There is a substantial similarity between the zebrafish and these other species in terms of the temporal and spatial expression pattern of *atoh8* during embryogenesis [9], suggesting that the gene serves a similar function in embryonic development in all these species.

Many members of the bHLH gene family, such as *Hes1, Hes5, Mash1* and *Math5*, are involved in Notch-Delta, Wnt and Nodal signal pathways which are known to be vital during retinogenesis [17], a process that involves the differentiation of different types of retinal cells in a fixed temporal sequence. Retinal ganglion cells (RGC) are the first to differentiate followed by bipolar cells and photoreceptors. Lastly, Müller glial cells are generated [18]. Particularly during the generation of RGC and phororeceptors, this process is regulated by transcription factors of the bHLH family. Inoue et al. demonstrated that Mis-expression of Math6 with retrovirus in the developing mouse retina induced neurogenesis, while inhibiting gliogenesis [9]. This is in agreement with our current findings that knockdown Atoh8 expression caused reduced number of ganglion cells in the retina. Further, substantial apoptotic cells were also seen in the retina, suggesting that the Atoh8 is also important in survival of neuronal cells. It was reported by Jean-Marc Matter et al. that cells with upregulated levels of *ath5* expression initiate transcription of early RGC-specific traits, exit the cell cycle and express Neuro M as well as other post-mitotic RGC-specific genes. Moreover, it was also reported that photoreceptor genesis involves two transcriptional pathways: *ngn2*→*neuroD*→*raxL* and *ath5*→*neuroD*→*raxL* [19]. Among these genes, *ngn2* and *ath5* function in progenitors and are responsible for cell type differentiation in vertebrate retina. *NeuroD* plays a central role in photoreceptor development. In the present study, we found that the expression of *ath5* was significantly reduced in the retinas of Atoh8 knock-down zebrafish, suggesting that Atoh8 might be the upstream regulator of *ath5* in the retina.

Atoh8 knock-down also resulted in distinct muscle abnormality. The malformations started at a very early developmental stage and most of the abnormal embryos shared the same phenotype. More importantly, we are the first to show that down regulation of *atoh8* expression resulted in changes of the master myogenic transcription factor *myoD* in early myogenesis of zebrafish, indicated that *atoh8* might play a crucial role in zebrafish during the early somite formation. Coutelle et al. found that Hh signaling was required for the expression of *myoD* and *myf5* as well as for differentiation in zebrafish adaxial myogenesis [20]. It remains to be investigated whether *atoh8* participates in the Hh signaling pathway during development somite and the detail function of *atoh8* in development of somite is also not clear.

It was suggested that *atoh8(Math6)*-null mice might be embryonically lethal [11] because of the absence of homozygous embryos. This is in consistent with the high mortality and substantial retinal cell apoptosis seen in zebrafish embryos treated with Atoh8*-*MO. Given the high level of expression and wide distribution of *atoh8* during early embryonic zebrafish development, it would not be surprising if this gene is involved in regulation of the expression of many downstream genes and also has an effect on the formation of many other organs besides retina and somites. For instance, evidence of heart defects, including altered jogging, abnormal expansion and pericardial edema, was seen in about half of the Atoh8 knock-down fish (data not shown).

To our knowledge, the present study is the first to demonstrate roles of endogenous *atoh8* in early embryonic development in vertebrates. It is also the first time that *atoh8* is found to regulate somite development. Our results suggest that *atoh8* may be involved in many aspects of early embryonic development and that zebrafish is a good model system for studying the function of *atoh8* in signal pathways governing cell differentiation in various organs.

## Materials and Methods

### Zebrafish maintenance

Zebrafish (AB strain) were maintained at 28.5°C on a 14-hour light/10-hour dark cycle as described by Westerfield [21]. Embryos were staged according to their morphological features and hours (h) or days (d) post-fertilization as described by Kimmel et al [22]. Embryos used in whole-mount *in situ* hybridization were cultured in Holtfreter solution with 0.003% phenylthiourea (PTU) (Sigma).

### Cloning and analysis of the zebrafish *atoh8* gene

To clone the *atoh8* cDNA, total RNA was isolated from zebrafish embryos (36 hpf) using a Qiagen miniprep procedure. PCR amplification was carried out with primers 5′-CGGGATCCTGGACTTATTAGTCAGGCTGGA-3′ (forward) and 5′-GGAATTCGAGGTCCTTCTTGCTCCAGAT-3′ (reverse) using the above cDNA as the template. The amplified product was cloned into pGEM-T Easy Vector (Promega) and sequenced. The full-length cDNA of zebrafish *atoh8* was submitted to GenBank under accession number EU272033. To determine online the predicted amino acid sequences of *atoh8* and conserved domains, we used National Center for Biotechnology Information (NCBI) Blast Server 2.0 to create multiple alignments and ClustalW version 1.82 (http://www.ebi.ac.uk/clustalw/) to construct the phylogenetic trees.

### Whole-mount *in situ* hybridization

To study the expression pattern of the *atoh8* gene during embryogenesis, the embryos were fixed in phosphate buffered saline with 4% paraformaldehyde (PFA) and were then processed for whole-mount *in situ* hybridization as described by Westerfield [21]. The zebrafish *atoh8* cDNA was cloned into pGEM-T easy vector, which contained T7 and SP6 promoters. The plasmid DNA was linearized with ApaI, and transcribed in vitro by T7 and SP6 RNA polymerases for the antisense and sense *atoh8* RNA probe. This was labeled with digoxigenin (DIG) (Roche) and pGEM-T-*atoh8* was used as the template. Other antisense RNA probes used in this study included *myoD* [23], *myf5, myogenin* (from P.J. Zhang), *Pax6a* and *ath5* (provided by D. L. Stenkamp and R. Frey).

### Morpholino oligo and RNA injection

Translation blocking morpholino antisense oligonucleotides (Atoh8*-*MO1, 5′-TGTTTAGATGTGGGTTCTTCATTTC-3′) and splicing blocking morpholino antisense oligonucleotides (Atoh8splI1E2 5′-GTACCTGTAAAAGTAGATCAAAGGG-3′) were designed and synthesized by Gene-Tools LLC. Mismatched MOs (Atoh8-MO1mis 5′-TGATTACATGTGGCTTCTTGATATC-3′, and Atoh8spmis 5′–GTACGTCTATAAGTACATCAAACGG-3′ were used as controls for Atoh8*-*MO1 and Atoh8splI1E2, respectively. Moreover, a *p53*-MO with the sequence 5′-GACCTCCTCTCCACTAAACTACGAT-3′ was also purchased from Gene-Tools, LLC.

The above morpholinos were injected into the zebrafish embryos at one cell stage with the *p53*-MO at a concentration ratio of 1∶1.5 (1.5 for p53-MO). The latter is to rule out the possibility that the effect of Atoh8-MO was mediated through a general p53-induced apoptosis [12]–[13].

For rescue experiment, the coding sequences of zebrafish atoh8 were cloned into the expression vector pcDNA3.0. The capped mRNAs were synthesized in vitro using EcoR I linearized plasmid DNAs and the T7 promoter. When coinjection of mRNA with the MO was performed, the embryos was first injected with the mRNA and then with the morpholino at a dose described in the text.

All images were captured using an IP-20 (OLYMPUS) under an IX-12 (OLYMPUS).

### Real-time quantitative PCR

The total RNA of control embryos and MO-treated embryos were extracted using Trizol(Invitrogen) method. For each sample the same quantity of total RNA (1 µg) was reverse transcribed into cDNA using the SMARTer™ PCR cDNA Synthesis Kit (Clontech). The real-time PCR was performed using SYBR® Green (Biotium) and 2 µl of cDNA template with RG-3000 real-time PCR apparatus (Corbett). Each individual reaction was performed in triplicate. A set of primer sequences for target genes were designed using Primer Primer5.0 and the sequences were sense: 5′ CAGGCAAGTCCTCAAGTGTC 3′, antisense: 5′ TGCGGTGGTCTGGTGTAT 3′, respectively. The PCR product was 379bp(373∼751 in *atoh8* mRNA). A set of control primers (β-actin) were used to normalize the abundance of cDNA in each reaction. The condition for Real-Time PCR was 95°C 5 min, then 95°C 10 sec, 60°C 15 sec, 72°C 30 sec for 40 Cycling, Melt: from 72°C to 99°C. For each comparison we calculated a 95% confidence interval about mean fold change, based on the expression level estimates across the 5 experimental replicates.

### Cryostat sectioning

Embryos fixed in PFA were embedded in 1.5% agarose/5% sucrose [24]–[25], incubated overnight in 30% sucrose and sectioned at 5–7 µm using a SME620 (Thermo Shandon).

### TUNEL assay

Embryos were fixed as for *in situ* hybridization and keep in methanol. After rehydration, embryos were sectioned and then permeabilized by proteinase K digestion, re-fixed in 4% PFA and washed in TBS. Apoptosis was detected in embryos by terminal transferase dUTP nick-end labeling (TUNEL), as described in the manufacturer's instructions (TdT-FragEL™ DNA Fragmentation Detection Kit: DAB, Merck).
